# Why Do Some Spanish Nursing Students with Menstrual Pain Fail to Consult Healthcare Professionals?

**DOI:** 10.3390/ijerph17218173

**Published:** 2020-11-05

**Authors:** Juan Diego Ramos-Pichardo, Ángela María Ortega-Galán, María Teresa Iglesias-López, Ana Abreu-Sánchez, Elia Fernández-Martínez

**Affiliations:** 1 Department of Nursing, University of Huelva, 21071 Huelva, Spain; juan.ramos@denf.uhu.es (J.D.R.-P.); angela.ortega@denf.uhu.es (Á.M.O.-G.); abreu@denf.uhu.es (A.A.-S.); 2 Faculty of Health Sciences, Universidad Francisco de Vitoria, Crta. Pozuelo-Majadahonda km 1800, Pozuelo de Alarcón, 28223 Madrid, Spain; m.iglesias.prof@ufv.es

**Keywords:** dysmenorrhea, menstrual pain, qualitative research, pain management

## Abstract

Dysmenorrhea is a problem that affects a large percentage of young women worldwide. Alarmingly, the majority of these women choose to self-medicate rather than consult a healthcare professional, despite the risks involved. The present study aimed to explore the reasons why undergraduate nursing students do not consult health care professionals regarding their menstrual pain. A qualitative study was conducted using an open question: “Why didn’t you consult a healthcare professional?” within the context of a research project on primary dysmenorrhea among nursing students at the University of Huelva, Spain. The responses of 202 women were analyzed using content analysis. Three categories were identified: assessment of the pain experienced, expectations, and experiences of professional care and selfcare. We found a striking normalization of the problem; notably, students downplayed the importance of the problem, considering that it was not worth consulting a physician. Furthermore, there was a notable degree of self-medication using non-steroidal anti-inflammatories (NSAIDs). These results may be useful for orienting policies to raise social awareness of this problem and for designing health education strategies aimed at women with primary dysmenorrhea.

## 1. Introduction

Menstrual pain or dysmenorrhea is a problem affecting a large number of women of reproductive age. It is estimated that 50 to 90% of female university students globally, and approximately 75% of Spanish female university students [1,2] suffer from this problem. The main symptom is acute menstrual pain, frequently accompanied by other symptoms such as gastrointestinal disorders, dizziness, irritability, depression and bloating [2,3]. Two types of dysmenorrhea have been identified: primary and secondary. Primary dysmenorrhea is not caused by any identified organic cause, whereas secondary dysmenorrhea is associated with other pathologies, such as endometriosis [4,5].

The physiopathology of primary dysmenorrhea has been the subject of numerous studies; the main theory is currently based on an increase in the level of prostaglandins [1], although the immune, neuroendocrine and vascular systems are also involved [5,6,7]. A number of studies have linked primary dysmenorrhea to a decrease in quality of life, as well as absenteeism and attending work despite feeling ill during university education, which may negatively impact academic performance and have significant socio-economic consequences [6,7,8].

Several pharmacological and non-pharmacological treatments have proven to be effective for dysmenorrhea [9], however, approximately 80% of Spanish female university students self-medicate as a pain management strategy, mainly relying on non-steroidal anti-inflammatories (NSAIDs), and rarely seek professional medical advice [6]. Self-medication usually consists of over-the-counter drugs such as NSAIDs for chronic pain management, which is currently considered a public health problem [10]. Specifically, women who suffer from dysmenorrhea usually chose the correct drug, however, the dose is usually subtherapeutic and approximately 20% of women with this problem are resistant to this type of analgesic. Therefore, this usually leads to inadequate pain control [11,12]. Although there are several non-pharmacological methods with proven efficacy for reducing dysmenorrhea, such as local heat or exercise, the women in our study chose to use non-pharmacological methods for which scientific evidence is still limited [13,14].

In addition, not consulting a health professional in the event of menstrual pain may result in delayed diagnosis of secondary dysmenorrhea, which displays similar symptoms and is often caused by endometriosis [15,16]. An early medical consultation reduces complications. For example, in the case of secondary dysmenorrhea produced by endometriosis, early diagnosis is considered essential to reduce the physical, emotional and social side-effects of this condition [17,18].

Notably, the delayed diagnosis and treatment of other problems among young women who present with pelvic pain, such as infections, can impact their future fertility [19]. Moreover, the risks and possible harmful side effects of self-medication, such as those related to the onset or worsening of gastrointestinal problems, should not be overlooked [11,20,21,22,23]. Furthermore, dysmenorrhea has been related to psychological disorders such as stress, anxiety and depression, and therefore the performance of a psychological assessment before choosing therapeutic methods may be necessary [24]. In addition, there is evidence that early medical assessment is more cost-effective than self-care in cases of dysmenorrhea [17]. Along these lines, the Spanish Society of Gynecology and Obstetrics (SEGO) advises young women to consult a health professional when faced with menstrual pain. This is in accordance with the informative leaflets aimed at young women, although referral to consult a specialized gynecologist is only indicated in cases of severe intensity or when pain is accompanied by other characteristics or symptoms [25,26].

In recent years, researchers have begun to conduct qualitative studies on aspects related to primary dysmenorrhea. The studies by Chen et al. [27,28] on the opinions of American women with dysmenorrhea and the reasons they decided to not consult a healthcare professional, found that most women assume that pain is the norm, or they prefer to self-manage symptoms. Some even consider that professionals have limited resources to help them, or that providers may not offer help. Other women feel too embarrassed or afraid to seek care, sometimes because they do not seek health care in general. Several authors highlight the variability of cultural and social opinions and attitudes about menstruation, which has traditionally been surrounded by multiple myths and taboos and continues to condition women’s attitudes today [29,30]. Along these lines, Tan et al. recognized that effective management of menstrual problems implies a biocultural approach based on culturally sensitive therapies [31].

Another relevant aspect is the organization of the health care system, which differs significantly between territories. In Spain, health care is public and free of charge, and, in the event of non-urgent consultations, users are attended by a primary care physician or a nurse with a short waiting list, within 48 h at the centers where tele-medicine systems have been implemented [32]. However, access to a gynecologist is only possible free of charge if indicated by the primary care physician, or privately at the patient’s own expense [33].

Several studies to date have examined the management of menstrual pain and problems surrounding self-medication, primarily among young women [2,17,34]. However, a knowledge gap exists in Spain regarding why women from different regions fail to consult health professionals for these issues. Considering the socio-cultural and health variability across different territories, it is interesting to explore this issue in each context, in order to design effective health strategies. Therefore, the aim of this study was to explore the reasons why undergraduate nursing students do not consult health care professionals regarding their menstrual pain.

## 2. Materials and Methods

### 2.1. Study Design and Participants

The data presented in this article correspond to the first phase of a broader project on dysmenorrhea among Spanish university students.

The eligibility criteria were: (1) being over 18 years old, (2) enrolled in the academic year 2018/2019 and 2019/2020 at the Nursing Faculty of the University of Huelva, (3) self-identified as having experienced symptoms of dysmenorrhea in the last six months [28,35], (4) having experienced pain during at least three menstrual cycles in the past year [2]. Women who were not in the classroom at the time of the invitation to participate were excluded.

The sample analyzed in the present study, based on a purposeful sample [36], was composed of participants who also met two additional criteria: (1) they stated that they had not consulted a health care professional regarding their menstrual pain and (2) they answered the open-ended question regarding the reasons for this decision.

Of the 514 students invited to participate in the project, 362 (70.4%) met the general inclusion criteria and were therefore included in the project. Of these, 55.8% (*n* = 202) participants also met the specific criteria of this study and were intentionally selected. It should be noted that all the women who stated that they had not consulted a professional answered the open question posed in relation to the reasons for not consulting a professional.

### 2.2. Data Collection

The invitation to participate in the study took place in the students’ faculty classrooms during the months of September and October 2019. The lead researcher visited the classroom at the end of the day and explained the purposes of the study to the group. Participation was voluntary and was not linked to any academic evaluation or course content. Students who agreed to participate were asked to complete a form with their personal data, including sociodemographic information, and questions concerning sociodemographic issues (age, area of residence, whether they worked during their studies, and whether they had studied the subject of women’s health) and gynaecological issues (age of menarche, number of children, cycle regularity, cycle length and menstrual flow, duration of menstrual pain, intensity of menstrual pain using the visual analogue scale (0–10), first degree family member affected, consumption of analgesics, oral contraceptives (OCPs) and non-pharmacological methods for the relief of menstrual pain).

In addition, college students who suffered from menstrual cramps throughout most of their periods were asked whether they had sought professional help via a closed-ended question (Have you ever gone to a healthcare professional for this pain?). Those who answered “no” were asked about their reasons for this behaviour using an open question (Why didn’t you consult a healthcare professional?”). We collected the data using an open question, in line with other studies on similar subjects [27,28].

This study was approved by the research ethics committee of Andalucía. In addition, all students provided consent prior to participating in the study.

### 2.3. Data Analysis

For the description of the sample, a bivariate descriptive analysis was carried out comparing the participants who met specific inclusion criteria for the present study (not having consulted a health professional and answering the open question about the reasons for not doing so) and those who did not meet the specific criteria despite meeting the general project criteria. For this purpose, the Mann-Whitney chi-square test and Mann-Whitney U was used. The significance level was set at 0.05.

In relation to the principal aim of this study, a content analysis of the responses given by the participants was conducted following the steps indicated by Graneheim and Lundman [37]: two experienced researchers (J.D.R.P. and E.F.M.) individually read the responses to obtain an overview of the answers. Subsequently, both researchers individually read the responses, taking note of the excerpts of text (words or phrases) which referred to the reasons why the respondents failed to seek professional care (identification of meaning units). Discrepancies between the researchers were resolved in meetings and a consensus was achieved thanks to the participation of a third researcher (A.O.G.) with greater experience in qualitative analysis.

The meaning units were labelled and grouped in codes according to their content. These codes were compared according to their similarity, and subsequently discussed and reviewed by the researchers, reaching a consensus on the final grouping of the codes into categories. Finally, through an interpretive process via group meetings (J.D.R.P., E.F.M., A.O.G. and A.A.S.) the categories were grouped into the three identified themes. The ATLAS.ti7 tool (version 7.0, ATLAS.ti Scientific Software Development GmbH, Berlin, Germany) was used for data analysis.

### 2.4. Trustworthiness

To ensure the credibility, reliability, confirmability and transferability [38] of the data, different strategies were employed: two researchers participated in the identification of meaning units, consensus meetings took place between researchers during the coding and grouping process and the selection of a broad sample of university students. Additionally, the final results were shared with 10 participants to verify their overall agreement with the results.

## 3. Results

### 3.1. Sociodemographic Characteristics of the Sample and Comparison with Excluded Participants

Table 1 describes the main characteristics and gynecological profile of the sample studied. The mean age was 21 years, 80.2% lived in an urban setting, 10.9% worked while completing their studies and 52.4% had already completed a course related to women’s health during their nursing studies. This table also presents a comparison with the students who, even though they met the general criteria for participating in the project, were excluded from this study because they had consulted a health professional regarding their condition and therefore did not meet the specific criteria. Overall, the participants included in the study sample were slightly younger, had a less frequent consumption of OCPs and had a slightly lower mean number of pain days per cycle and lower intensity of menstrual pain.

### 3.2. Why Do Women Fail to Consult Healthcare Professionals?

After analyzing the responses, a total of 290 meaning units were drawn from the answers of the participants to the open question. These were grouped into 11 categories and, in turn, classified into three principal themes: Underestimation of pain, Low expectations regarding health care and Prefers self-care (Table 2).

#### 3.2.1. Underestimation of Pain

Table 3 presents the relationship between categories and codes as well as providing some examples of meaning units in relation to the topic “underestimation of pain”.

The majority of participants considered their pain a normal part of menstruation, even natural:

P4 … I think it’s normal, it’s just the way it is.
P58 … it’s part of the period.
P176 … it’s a normal symptom of the period.

Some women considered menstrual pain as something that happened to most women. This view of normality had been instilled by their closest family and they had never considered it to be a problem until their participation in the study.

P40 … it happens to everyone.
P44 My whole family suffers menstrual pain and they consider it normal.
P148 most women suffer from it.

Additionally, many considered the pain to be bearable, either because of its limited intensity or duration, and so did not consider it to be a cause for concern.

P37 The pain doesn’t last long.
P95 It’s only 1 or 2 days every period and I have to deal with it.

#### 3.2.2. Low Expectations Regarding Health Care

Table 4 summarizes the categories and codes for this topic and includes some examples of meaning units. Given that the pain was considered normal or not serious, the university students reported that the pain was not a valid reason to see a healthcare professional.

P85 I don’t think it’s a good reason.
P137 Because I don’t think it’s serious enough to go to a health clinic.
P119 …as long as the pain is not too bad I don’t think it’s necessary to see a doctor.
P200 It’s not serious enough for me to need to see a healthcare professional.

Some participants reported having seen a healthcare professional in the past and that the experience did not meet their expectations:

P147 I went to the emergency room for the pain and they gave me an analgesic and didn’t give it any importance…

Others predicted that the medical attention, treatment or advice they would receive would be insufficient and chose to avoid feelings of disappointment or disagreement. The treatment that most participants expected to receive was anti-inflammatory analgesics and/or hormonal therapy:

P57 …they generally just give anti-inflammatories.
P89 Because the most probable answer I’ll get is a hormonal contraceptive and I don’t want that kind of treatment.
P193 They always say it’s normal; they give you analgesics and send you home.

Others reported their conviction that a professional would not be able to help them with this problem or even expressed feelings of distrust, shame or discomfort when seeing a professional.

P8 Because it’s a bit uncomfortable.
P36 …a doctor won’t be able to do much.
P83 …I feel a bit ashamed that if I see a doctor they will just trivialise the problem.

Finally, some women reported not seeking professional care because of a lack of time.

P41 I don’t have time.
P91 I don’t have time to see a gynaecologist and getting an appointment takes forever.

#### 3.2.3. A Preference for Self-Care

Finally, the third theme that emerged from the responses referred to the practice of selfcare to deal with menstrual pain and is presented in Table 5. The most common practice, described as “self-medication”, is the use of non-steroidal anti-inflammatories (NSAIDs), although some participants mentioned hormonal contraceptives:

P11 The pain is easier because I take oral contraceptives.
P38 The pain generally goes away with an anti-inflammatory.
P126 I mange with an analgesic.
P202 I deal with the pain using analgesic medication.

Some participants also mentioned the use of local heat as a non-pharmacological method for relieving the pain of dysmenorrhea.

P37 I also use physical measures like local heat to relieve the pain.
P108 …I need to be in bed and apply local heat for a few hours.

Several participants also referred to the need to simply “put up with” the pain without doing anything or seeking help to manage the pain.

P4 … it’s just the way it is.
P65 … just put up with the pain until I can’t take it anymore.

## 4. Discussion

The aim of this study was to explore the reasons why undergraduate nursing students do not consult health care professionals regarding their menstrual pain. By identifying the origin of this problem, often resulting in self-medication, decreased quality of life, increased absenteeism and delayed diagnosis of other pathologies, this may help focus public health strategies on how to best approach this problem. The main results can be summarized by the fact that future Spanish nurses normalize menstrual pain and underestimate it, have a lack of confidence in health professionals when consulting menstrual issues and express a preference for self-care, principally by self-medicating with NSAIDs.

The normalization of dysmenorrhea has been described in previous international studies, including a study by Wood et al. among university students in Pennsylvania, a paper by Brantelid involving Swedish women of diverse academic backgrounds and studies by Chen et al. among American women [28,35,39,40]. However, to date, there have been no studies conducted in Spain. In particular, a striking finding were the repeated statements referring to menstrual pain as being normal and natural, despite the fact that the participants in our study were future health care professionals receiving training in women’s health issues and who are assumed to have more gynecological knowledge than the average person. This may be attributed to deep cultural factors in Spanish society, in a culture where women are expected to bear this natural pain [41]. Our results show that family and close personal relations are particularly important cultural factors in the normalization of this problem. This aspect is particularly the case when considering that previous studies have shown that having an immediate family member with dysmenorrhea is a risk factor for suffering this problem; it is therefore typical for various family generations of the same family to have the same problem [42,43,44]. This cultural influence and socially imposed gender roles may lead to learned behavior in dealing with this pain, which women believe they must simply bear as something implicit to womanhood. 

Dysmenorrhea is considered an illness according to the International Classification of Diseases (ICD) and some countries, such as Japan, Indonesia and South Korea have enacted specific legislation regarding medical leave for this reason [45]. In Spain, there are no specific regulations, however, it is recognized as a temporary disability justifying sick leave [46]. In contrast, the future healthcare professionals participating in this study, despite suffering from this ailment first-hand, do not regard it as a sufficient reason to consult a healthcare professional. This finding indicates that the physiopathology and the reasons for consultation for menstrual alterations are an area that should be studied in depth during undergraduate nursing training, as it is likely that, if they maintain the perspective of normalization they will also apply this during their professional practice when caring for other women. This issue should be explored in depth in future studies by analyzing whether health professionals themselves also tend to normalize this problem.

The expectations that young women have regarding health care professionals are low. Some participants even reported that feelings of shame and discomfort dissuaded them from seeking medical care. These results are in line with those of other researchers, such as Chang et al. among Chinese women and Tanakaa et al. in a sample of Japanese women [47,48]; although these studies were centered on a younger female population. It is particularly striking that, in our case, these types of responses came from university students studying to be future health care professionals. The feelings of shame and discomfort may also be to a fear of being stigmatized for suffering a psychosomatic illness, given the stigmatization of those suffering from mental health disorders even on behalf of health care professionals [49,50]. These feelings may also be associated with a possible complex about being considered more sensitive to pain than other women, as some studies indicate that women suffering from dysmenorrhea have greater sensitivity to pain in general [1,51].

Participants in this study expressed their preference for self-care, principally by self-medicating with NSAIDs. In this sense, the different positions found in the medical literature regarding primary dysmenorrhea and its professional treatment is striking. This includes information about gynecological issues aimed at young women containing contradictory messages, often normalizing or minimizing the importance of menstrual pain. These messages undoubtedly influence the decision of the majority of women with dysmenorrhea not to seek medical help. For example, a public message from the Spanish Society of Obstetrics and Gynecology (SEGO), available online, promotes self-medication, recommending girls to take anti-inflammatories for menstrual pain, without previously consulting a physician [52]. This contradicts another public health message by the same organization, which recommends seeing a gynecologist in the case of menstrual pain [26]. Our results support the need for women to be informed and empowered by health care providers to make appropriate decisions about their own menstrual health.

A comprehensive comparison of the results of the current study with the only study of similar characteristics identified in the literature, carried out by Chen et al. on American women, reveals that this former study identified nine categories of reasons acknowledged by women: assuming symptoms are normal, preferring the self-management of symptoms, limited resources, thinking that health professionals would not be able to offer help, lack of awareness regarding treatment options, considering their symptoms to be tolerable, being wary of available treatments, feelings of embarrassment or fear, and not seeking health care in general [28]. In contrast, the present study identified four main themes. The main differences between the results of both studies are that three issues were identified in American women that were not present in Spanish women: having limited resources, being unaware of treatment options, and not seeking medical care in general. Regarding the latter, although it was not identified as such as a topic or category in our study, some participants did state that they avoided consulting health professionals, which is even more striking considering they are students of health sciences, suggesting that it would be interesting for future studies to explore why some people avoid consulting health professionals. Regarding the other differences between these studies, these could be due to socio-cultural factors, however, they are fundamentally justified by the differences in the access to the health system in both contexts. Thus, in Spain, access to health care is free with short waiting times for attendance by a primary care professional [39], compared to the US healthcare system. Furthermore, the sample analyzed by former studies included women of different ages and academic levels, unlike the present study which only included female university students studying health sciences. Thus, it seems coherent that in the present study we did not identify “lack of knowledge regarding treatment options” as our sample is more likely to have greater skills and access to information about medical treatments. Nonetheless, it seems that the normalization of pain, the belief that symptoms should be tolerated, feelings of fear/shame and distrust towards health care providers and the belief that they will not help, were findings that arose in the stories of women in both studies and are likely to reflect a strong cultural influence based on fears and taboos regarding menstruation [29,30], fear of stigmatization [53] and concern about lack of sensitivity on the behalf of health care providers towards the problems they face, which is more striking among future nurses.

The principal limitation of the study is that the information was gathered based on a single open-ended question which did not allow for follow-up questions to be asked for more in-depth understanding; however, this type of data collection and analysis was also applied for the same purpose by Chen et al. in a cohort of American women with dysmenorrhea [13]. Another limitation was that the sample was drawn from students of only one nursing school. Due to the type of sampling proposed, there was no content saturation. In addition, some variables were not collected, such as socioeconomic level and number of pregnancies, which may have provided further valuable information. By qualitatively analyzing a sample of women who did not consult health professionals about their condition, we missed the opportunity to explore the reasons why women do consult health professionals. Finally, some psychological variables that have been related to dysmenorrhea, such as stress, anxiety and depression [24], were not taken into consideration in the present study and therefore it would be interesting to analyze women’s attitudes in future studies with this in mind. The strengths of this study design include the participation of large numbers of women with similar characteristics, in this case, nursing students at a Spanish university suffering from primary dysmenorrhea who do not seek professional healthcare advice for this problem.

**Figure 1:**
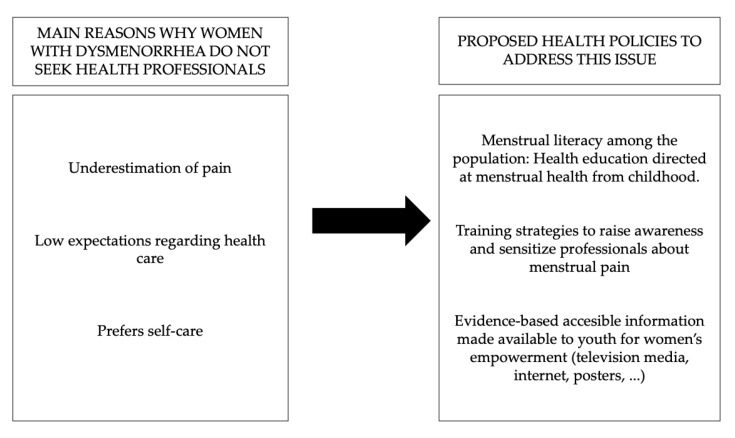
Main reasons why women with dysmenorrhea do not seek health consultations and proposed health policies to address this issue.

Based on the results of this study, it would be interesting to pursue this line of research, developing and implementing educational strategies on women’s health issues. Additionally, it would be interesting to implement changes in health policies. Figure 1 reflects the suggested proposal, which includes promoting menstrual literacy in schools, implementing strategies to raise awareness and sensitize professionals about this problem, and enhancing the dissemination of evidence-based information made accessible through different media to promote women’s empowerment. These findings also invite an exploration of the scepticism and distrust towards healthcare professionals expressed by the participants and the feelings of shame or discomfort in the case of menstrual pain, as well as an analysis, from the perspective of gender, of why many women believe they must simply “put up with menstrual pain”.

## 5. Conclusions

Most Spanish nursing students in this study stated that they considered menstrual pain to be something both normal and natural. The fact that the duration of the pain is limited in time and is alleviated by self-medication with NSAIDs also contributes to the reluctance to consult a healthcare professional. Furthermore, this attitude is also supported by the low expectations or negative experiences of treatment from healthcare professionals. The results of this study may be useful for guiding policies to raise social awareness of this problem and for the design of health education strategies aimed at women with primary dysmenorrhea to empower women on the subject of menstruation from childhood. Furthermore, these findings will enable professionals to become aware of the need for greater awareness and to approach the young female population by discussing these issues.

## Figures and Tables

**Table 1:** Comparison of the characteristics of the women who consult health professionals and those who chose not to do so.

Variables		Excluded Sample (Professionals Were Consulted) (*n* = 160) *n* (%)/M ± SD	Study Sample (Professionals Were not Consulted) (*n* = 202) *n*(%)/M ± SD	*p*-Value
Age (years)		22.0 ± 4.4	21.1 ± 2.4	0.031 ^a,^*
Weight (kg)		61.0 ± 9.5	60.3 ± 9.5	0.471
Height(cm)		164.5 ± 6.2	163.9 ± 6.3	0.400
BMI (kg/m^2^)		22.6 ± 3.3	22.4 ± 3.3	0.749
Residential setting	Rural	40 (50%)	40 (50%)	0.237 ^b^
	Urban	120 (42.6%)	162 (57.4%)	
Worked while studying	No	143 (44.3%)	180 (55.7%)	0.935 ^b^
	Yes	17 (43.6%)	22 (56.4%)	
Completed the ‘women’s health’ subject	No	69 (40.4%)	102 (59.6%)	0.163 ^b^
	Yes	91 (47.6%)	100 (52.4%)	
Age of menarche		12.1 ± 1.6	12.2 ± 1.5	0.814 ^a^
Regular cycle	No	53 (50%)	53 (50%)	0.153 ^b^
	Yes	107 (41.8%)	149 (58.2%)	
Days of bleeding		4.9 ± 1.2	5.0 ± 1.3	0.360 ^a^
Amount of flow	Low/Medium	120 (42.1%)	165 (57.9%)	0.123
	Abundant	40 (51.9%)	37 (48.1%)	
Cycle duration		29.7 ± 8.8	29.9 ± 7.3	0.903 ^a^
Days of menstrual pain		2.5 ± 1.2	2.1 ± 1.0	0.001 ^a,^*
First degree relative affected	No	67 (49.6%)	69 (50.4%)	0.096 ^b^
	Yes	91 (40.6%)	133 (59.4%)	
Intensity of menstrual pain (VAS)		7.0 ± 2.1	6.3 ± 1.8	0.002 ^a,^*
Self-medication with analgesics	No	47 (42.7%)	63 (57.3%)	0.768 ^b^
	Yes	111 (44.4%)	139 (55.6)	
Consumption of contraceptives (OCPs)	No	87 (37.2%)	147 (62.8%)	<0.01 ^b,^*
	Yes	73 (57%)	55 (43%)	
Use of non-pharmacological methods of pain relief	No	102 (45.7%)	121 (54.3%)	0.504
	Yes	56 (42.1%)	77 (57.9%)	

^a^ Student’s *t*-test, ^b^ Chi square Test; * *p* < 0.05.

**Table 2:** Structure of the principal themes and categories.

Principal Themes	Categories
Underestimation of pain	It’s normal
	It’s bearable
	It’s not a worry
	It doesn’t last long
Low expectations regarding health care	It’s not a good enough reason to see a doctor
	Disappointed expectations
	Lack of trust
	Lack of time
Prefers self-care	Self-medication
	Non-pharmacological methods
	Put up with the pain

**Table 3:** Categories, codes, and examples of the theme “underestimation of pain”.

Categories	Codes	e.g., Meaning Units
It’s normal	Normalisation	P13 I guess it’s normal to have pain. P32 I think it’s normal, since I’ve always had pain with my period. P85 … menstrual pain is normal and natural.
	Known cause	P100 Because I consider that it is a pain with a known cause, and although it is annoying and sometimes intense, I don’t go to the doctor.
	It also happens to my family	P96 My mother, my aunt and my sister also get it. It happens to all of us, and we’ve never been to the doctor because of it. P75 Because in my family it is considered normal.
It’s bearable	Bearable	P18 Because I consider it a bearable and endurable pain. P23 It’s bearable
	Low intensity	P16 It is not a very intense pain and I don’t need to consult a doctor. P185 It is not excessive or intense pain.
	Not at all worrying	P21 I assumed it was because of my period and I wasn’t too worried about it. P97 It’s painful but it’s not too extreme or worrying.
It’s not a worry	It’s not limiting	P138 Because it isn’t a disabling pain and does not condition my life excessively. P140 Because it doesn’t limit my ability to do things.
	It isn’t important	P34 I haven’t given it more importance, I thought that it was simply period pain. P127 Because I don’t consider it important.
It doesn’t last long	Variable duration	P161 I don’t think it’s extremely important because sometimes it lasts several days but sometimes just a few hours.
	Short duration	P173 Because it’s only a pain that occurs just before I get my period, a short time, and I haven’t considered it important. P124 It lasts a short time. I only have it the first day and sometimes the second.

**Table 4:** Categories, codes and examples of the theme “Low expectations regarding health care”.

Categories	Codes	e.g., Meaning Units
It’s not a good enough reason to see a doctor	No consultation needed	P82 I don’t think it could be due to any major problem requiring me to see the doctor P113 I don’t think it’s a reason to see a doctor
	I don’t usually go to the doctor	P2 I don’t usually go to the doctor much, and even less if it’s something unimportant like this.
Disappointed expectations	Previous experiences	P147 I went to the emergency room for the pain and they gave me an analgesic and didn’t give it any importance… P198 My family doctor is never really interested in any of my problems and so I don’t bother going.
	Predicting care	P168 Because what they are saying is that I should take birth control pills. P177 I expect they won’t think it’s important or will give me hormonal contraceptives.
Lack of trust	They can’t help me	P36 …a doctor won’t be able to do much P111 A health professional is not going to change the situation much. It‘s unavoidable.
	It’s uncomfortable/feelings of shame	P8 Because it’s a bit uncomfortable P83 …I feel a bit ashamed that if I see a doctor they will just trivialise the problem.
Lack of time	I don’t have time	P55 I haven’t had time to make an appointment, although I will do so in the future. P107 I made an appointment once but I was on my way to exams. I didn’t go to the appointment and I didn’t go back for another appointment.
	Appointment delays	P28 It takes so long to get an appointment that it’s not worth asking. P170 If you ask for an appointment, when they give it to you the pain has already gone away.

**Table 5:** Categories, codes and examples of the topic “A preference for self-care”.

Categories	Codes	e.g., Meaning Units
Self-medication	Self-medication using hormonal contraceptives	P11 The pain is more bearable because I take oral contraceptives. P66 I usually take oral contraceptives and that works well for me.
	Self-medication using analgesics	P42 I can deal with it if I take 1 or 2 Naproxen. P65 I usually take ibuprofen… P202 I deal with the pain thanks to analgesic medication.
Non-pharmacological methods	Local heat	P56 …I apply heat to the area and that relieves the pain. P108 …I need to be in bed and apply local heat for a few hours.
Enduring the pain	Endure the pain	P105 I ‘ve always thought that menstrual pain must be endured. P122 That’s the way it is, you have to suffer it every month.
